# Short-Term Supplementation with 100% Bilberry Products and Its Effects on Body Composition and Lipid Profile in Overweight/Obese Women

**DOI:** 10.3390/metabo15040218

**Published:** 2025-03-24

**Authors:** Marta Habanova, Maros Bihari, Radek Latal, Martina Gažarova, Petra Lenártová, Jana Pastrnakova, Jadwiga Hamulka

**Affiliations:** 1 The Institute of Nutritional and Genomics, Slovak University of Agriculture, 94976 Nitra, Slovakia; maros1307@gmail.com (M.B.) xlatal@uniag.sk (R.L.); martina.gazarova@uniag.sk (M.G.); petra.lenartova@uniag.sk (P.L.); info@vitamine.sk (J.P.); 2 Department of Human Nutrition, Institute of Human Nutrition Sciences, Warsaw University of Life Sciences (SGGW-WULS), 02-787 Warszawa, Poland; jadwiga_hamulka@sggw.edu.pl

**Keywords:** overweight, obesity, bilberry, juice, fibre, lipid profile, sdLDL, intervention

## Abstract

**Introduction**: Overweight and obesity are major public health concerns, often leading to increased cardiovascular risk. **Methods**: This eight-week interventional study examined whether regular consumption of two natural bilberry products could improve body composition and lipid profiles in overweight/obese women. A total of 30 participants (aged 50–60 years) were assigned to consume either 125 mL/day of 100% bilberry juice or 10 g/day of 100% bilberry fibre, while maintaining their habitual diets and lifestyles. **Results**: Although no significant changes were found in anthropometric parameters or blood pressure in either group, both interventions reduced low-density lipoprotein cholesterol (LDL-C) and increased high-density lipoprotein cholesterol (HDL-C). Surprisingly, total cholesterol (TC) levels rose in the bilberry juice group (from 6.41 ± 1.23 mmol/L to 6.94 ± 1.30 mmol/L (*p* < 0.001)), and in the fibre group (from 6.06 ± 1.39 mmol/L to 6.43 ± 1.05 mmol/L (*p* = 0.046)), likely due to elevated HDL-C (*p* < 0.001) overshadowing the drop in LDL-C (*p* < 0.05). Triglyceride (TG) levels did not change significantly and were still within the reference range. **Conclusions**: Notably, the bilberry juice group experienced a significant reduction in atherogenic small dense LDL (sdLDL) subfractions, suggesting a favourable shift in cardiovascular risk factors. These findings highlight the potential of bilberry-based products as a supportive strategy for improving lipid profiles in overweight/obese women.

## 1. Introduction

Obesity is a complex chronic condition characterized by excessive adipose tissue accumulation, which can adversely affect health. In 2022, an estimated 2.5 billion adults worldwide (43% of individuals aged 18 years and older) were classified as overweight, with 890 million (16%) meeting the criteria for obesity [1]. Obesity is frequently associated with detrimental metabolic, biomechanical, psychosocial, and economic consequences [2]. Obesity and its related comorbidities significantly impact quality of life and reduce life expectancy [3]. Among the most commonly reported risks associated with obesity are cardiovascular diseases (CVD), in particular coronary heart disease and heart failure [4,5,6,7]. Sex-specific differences in obesity may carry significant implications for cardiovascular health. Halland et al. [8] reported that in the FATCOR study, the majority of obese and overweight individuals exhibited subclinical heart disease. This condition may contribute to the poorer prognosis associated with obesity. Notably, the prevalence of subclinical heart disease was higher among women than men.

The risk of CVD was 40% higher in overweight women, 60% higher in women with overall obesity, and 30% higher in women with abdominal obesity compared to men [9]. Increased overall adiposity and abdominal fat are associated with multiple cardiovascular pathological disorders, manifested in electrocardiographic, hemodynamic, structural, and functional changes [10].

Atherogenic dyslipidemia is strongly associated with obesity and plays a significant role in the increased risk of atherosclerosis and coronary artery disease. This dyslipidemic condition is characterized by high plasma concentrations of triglycerides (TGs) and apolipoprotein (apo) B-containing lipoproteins and low concentrations of high-density lipoprotein (HDL) [11]. Atherosclerosis, the primary cause of CVD, is linked to chronic inflammation and oxidative processes that result in the modification of atherogenic lipoproteins [12]. The atherogenic profile, marked by increased levels of VLDL, IDL 1-3, small HDL, and particularly high concentrations of small dense LDL (LDL 3-7) subfractions, may elevate the risk for atherogenesis and cardiovascular diseases (CVD). Elevated small dense lipoproteins (sdLDL) particle levels are associated with an increased risk of CVD [13,14]. These particles easily penetrate the subendothelial space and attach to the arterial wall, thereby increasing the risk of atherosclerosis [15]. To improve risk factors associated with dyslipidemia, a dietary strategy focused on heart-healthy eating habits is recommended, emphasizing plant-based foods including vegetables, fruits, whole grain products, legumes, and nuts [16]. These foods contain phytochemicals or produce secondary metabolites such as polyphenols, which may be considered non-essential nutrients with medicinal importance [17]. Polyphenols have demonstrated the ability to improve endothelial function, prevent abnormal platelet aggregation, reduce inflammation, and enhance the plasma lipid profile, all contributing to better cardiovascular health [18]. Several studies investigating the consumption of berries or berry-based products rich in bioactive compounds have demonstrated statistically significant positive effects on health when consumed regularly. These effects, particularly of bilberry consumption, include notable improvements in cardiovascular health parameters [19,20,21,22,23], showing substantial benefits [20,24].

Bilberry (*Vaccinium myrtillus* L.) is recognized as a functional food, with its fruits resembling small, dark blueberries (*Vaccinium corymbosum* L.). The distinctive colouration of bilberries is attributed to their high anthocyanin content, which is also linked to their beneficial health effects [25]. However, no clinical studies have been conducted on the relationship between the intake of 100% bilberry fruit juice or bilberry fibre and the modulation of the lipid profile and sdLDL subfractions in overweight/obese women.

This study aims to test the hypothesis that regular, short-term consumption of 100% bilberry juice or bilberry fibre, both rich sources of polyphenols and other bioactive compounds, may have beneficial effects on the modulation of CVD risk factors (e.g., improvement of body composition parameters, increase in HDL-C, and reduction in TC, LDL-C, TG, and the atherogenic LDL 3-7 subfraction) in middle-aged women with overweight/obesity. To test this hypothesis, the female employees of Slovak University of Agriculture volunteered to regularly drink 100% bilberry juice or bilberry fibre daily for 8 weeks. Their body composition and lipid profiles (TC, LDL-C, HDL-C, and TG), LDL subfractions (1–7) (including lipoprotein indexes TC/HDL ratio and LDL/HDL ratio), and atherogenic index of plasma (AIP) were monitored before (pre) and after (post) bilberry product intervention. While studies exist on mixed berry interventions, those on pure bilberry juice/fibre intake with direct sdLDL measurements in overweight/obese women are still underreported.

## 2. Materials and Methods

### 2.1. Participants and Study Design

This investigation was designed as a single-arm pre–post intervention study [26] focusing on how daily supplementation with 100% bilberry products might affect lipid profiles and other cardiovascular risk markers in overweight/obese women. Practical limitations precluded the inclusion of a parallel control group. Volunteer recruitment procedures were adapted from methods published by Habanova et al. (2022) [20], with slight modifications to fit the current context. Women aged 50–60 years qualified for participation if they (1) had a body mass index (BMI) ≥ 25 kg/m^2^, classifying them as overweight (25–30 kg/m^2^) or obese (>30 kg/m^2^); (2) presented with at least one risk factor for cardiovascular disease, such as dyslipidemia (TC > 5.20 mmol/L, LDL-C > 3.4 mmol/L, HDL-C < 1.03 mmol/L, or TG > 1.70 mmol/L), elevated blood pressure (systolic ≥ 140 mm Hg or diastolic ≥ 90 mm Hg), or atherogenic small dense LDL particles (LDL 3-7); (3) maintained a stable body weight (±3 kg) for the previous three months; and (4) reported an alcohol intake below 30 g/day.

Exclusion criteria encompassed any chronic or acute conditions such as diabetes, thyroid dysfunction, or active liver disease, as well as any use of corticosteroids, lipid-lowering medications, or supplements. Individuals with food intolerances to polyphenols, ongoing participation in other dietary interventions, or lifestyle factors such as smoking or heavy alcohol consumption were also excluded.

All participants gave their informed consent after receiving detailed information regarding the potential risks of the intervention. The Ethics Committee from the Specialized Hospital of St. Svorad Zobor (Nitra, Slovakia), and the Slovak University of Agriculture (Institute of Nutrition and Genomics) approved the study protocol (Study No. 4/071220/2020), which adhered to the Declaration of Helsinki guidelines.

### 2.2. Intervention

**Figure 1:**
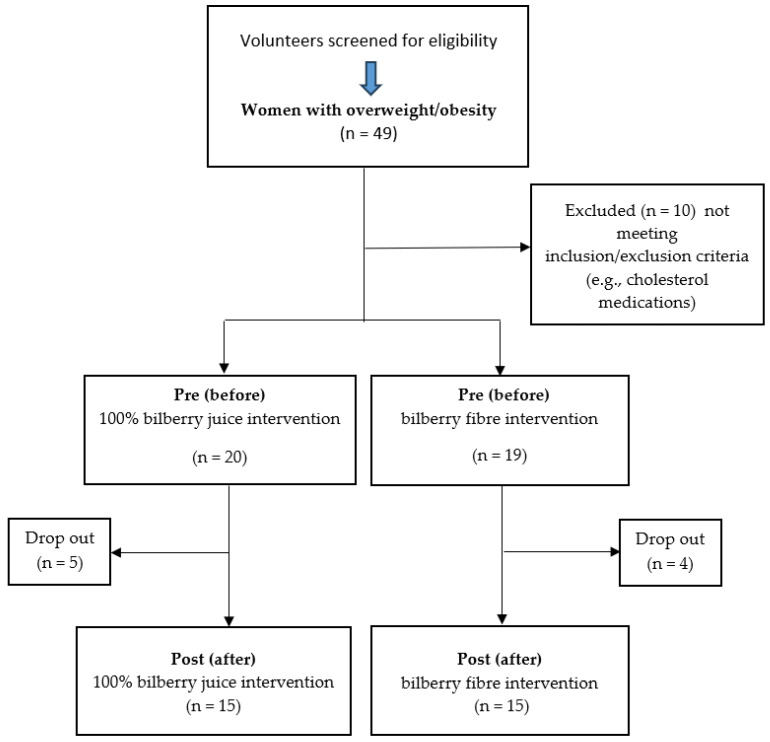
Flowchart: study design and sample collection. n = number of subjects.

Thirty overweight or obese women (mean age 55.83 ± 2.95 years) took part in an 8-week programme (see Figure 1). They were divided into two subgroups based on product allocation. One subgroup consumed 125 mL per day of 100% organic bilberry juice, while the other took 10 g per day of 100% organic bilberry fibre. Both products, manufactured in Slovakia by Wellberry, were produced exclusively from *Vaccinium myrtillus* L. with no additives. The juice underwent gentle pasteurization at 72 °C, while the fibre was derived from residual bilberry pomace after juice extraction. Participants were instructed to incorporate their assigned bilberry product into their usual diet without altering their normal eating habits or exercise routines.

To quantify key phytochemicals, antioxidant activity, and vitamin C in these bilberry products, standard analytical methods after their extraction were used. For the study, 1g of the sample was extracted with 20 mL of 80% ethanol for 24 h. The extract was then centrifuged at 4000 rpm for 10 min (Rotofix 32 A, Hettich, Kirchlengern, Germany). The supernatant was collected for further analyses, with extractions performed in triplicate. The Folin–Ciocalteu method described by Lachman et al. [27] estimated total phenolic content (in mg of gallic acid equivalents (GAE) per millilitre in juice and per gram in fibre), and a modified version of Lapornik et al. [28] was used to assess total anthocyanins. Specific phenolics were evaluated via HPLC-DAD (Agilent 1260 Infinity, Agilent Technologies, Waldbronn, Germany) based on adaptations of Gabriele et al. [29]. The DPPH assay [30] provided antioxidant activity results, and vitamin C content was measured on a Waters 2695 HPLC system (Waters Corporation, Milford, MA, USA) equipped with a UV–VIS spectrophotometer (Shimadzu UV-1800, Kyoto, Japan). [31].

The results were expressed in mg of gallic acid equivalents/mL for juice and in mg of gallic acid equivalents/g for dietary fibre. Total anthocyanins, caffeic acid, coumaric acid, ferric acid, rutin, myricetin, resveratrol and quercetin were expressed as mg/L in bilberry juice extract and as mg/g in dietary fibre of bilberry; vitamin C was expressed as µg/mL in bilberry juice extract and as µg/g in blueberry fibre. The percentage value of DPPH inhibition was calculated based on the following equation: DPPH inhibition (%) = [(A_0_ − A_10_)/A_0_] × 100, where A_0_ represents the absorbance at time *t* = 0 (DPPH solution), and *A*_10_ is the absorbance at time *t* = 10 min. All assays were conducted in multiple replicates, and results were reported as mean ± standard deviation (SD).

### 2.3. Anthropometric Measurements

At baseline (week 0) and after 8 weeks, trained staff performed anthropometric assessments under standardized conditions. Height (cm) was measured in a standing position using an electronic scale (Tanita WB-3000, Tanita Co., Tokyo, Japan), while body composition parameters—such as weight, body mass index (BMI), waist circumference, waist-to-hip ratio (WHR), skeletal muscle mass (SMM), fat mass (BFM), visceral fat area (VFA), and body fat percentage (PBF)—were determined using multi-frequency bioelectrical impedance (InBody 720, Biospace Co., Seoul, Republic of Korea). BMI categories were defined as follows: normal weight (18.5–24.99 kg/m^2^), overweight (25–30 kg/m^2^), and obese (>30 kg/m^2^) [31,32]. Blood pressure (BP) and heart rate were measured using a DM-3000 sphygmomanometer (Nihon Seimitsu Sokki Co. Ltd., Nakago Shibukawa, Gunma, Japan) while participants remained seated and rested for at least 15 min. Reference values for BP (<120/<80 mm Hg) followed American College of Cardiology/American Heart Association guidelines [33]. Each parameter was re-evaluated post-intervention under similar conditions to minimize variation.

### 2.4. Preparation of Blood Samples

Fasting venous blood (8 h overnight fast) was drawn from the cubital vein into two S-Monovette^®^ tubes (2.7 mL with EDTA and 7.5 mL with serum gel). Samples were then centrifuged in a Hettich^®^ MIKRO 220R (Andreas Hettich GmbH & Co., Tuttlingen, Germany) to separate plasma and serum. The EDTA tubes were spun at 1800 rpm for 15 min, and the serum gel tubes at 3000 rpm for 10 min, after which plasma and serum were stored at –80 °C until analysis.

### 2.5. Clinical Parameters

Lipid profile (total cholesterol, TC; triglycerides, TG; low-density lipoprotein cholesterol, LDL-C; and high-density lipoprotein cholesterol, HDL-C), fasting glucose, and additional parameters were analyzed on thawed serum samples using a BioMajesty^®^ JCA-BM6010/C automated system (JEOL Ltd., Tokyo, Japan). Commercial reagents from DiaSys (DiaSys Diagnostic System GmbH, Holzheim, Germany) and Randox (Randox Laboratories Ltd., Crumlin, UK) were used per the manufacturers’ protocols. Based on the resulting TC, TG, LDL-C, and HDL-C values, we calculated TC/HDL and LDL/HDL ratios [34,35], as well as the atherogenic index of plasma (AIP), defined as the log transformation of TG/HDL [36]. Lipoprotein subfractions (VLDL, IDL 1–3, large LDL [LDL1, LDL2], and sdLDL [LDL 3-7]) were identified with the Lipoprint^®^ system (Quantimetrix Corp., Redondo Beach, CA, USA) and interpreted as either atherogenic or non-atherogenic in accordance with Zitnanova et al. [37]. Measurements given in mg/dL were converted to mmol/L using the factor 0.0259. Pre–post comparisons followed the approach of Harris et al. [38], examining potential causal links to the intervention. 

### 2.6. Statistical Analysis

Data were processed with STATISTICA 13 (TIBCO Software, Inc., Palo Alto, CA, USA) and Microsoft^®^ Excel^®^ 2016. Normality was assessed with the Shapiro–Wilk test. Depending on the distribution, paired or two-sample t-tests and ANOVA (with post hoc Tukey’s test) were employed to identify differences between baseline and post-intervention values. Results are presented as mean ± standard deviation (SD), and a *p*-value < 0.05 denoted statistical significance.

## 3. Results

### 3.1. The Bioactive Compounds in 100% Bilberry Juice and Dietary Fibre of Bilberry

The concentrations of monitored bioactive compounds in bilberry juice and bilberry dietary fibre (including total phenolic content, total anthocyanins, caffeic acid, coumaric acid, ferulic acid, rutin, myricetin, resveratrol, quercetin, vitamin C, and antioxidant activity) are shown in Table 1. Some biologically active substances (such as ferulic acid, rutin, and vitamin C) were found in higher concentrations in bilberry juice. In contrast, others (such as total phenolic content, total anthocyanins, caffeic acid, coumaric acid, myricetin, resveratrol, quercetin, and antioxidant activity) were present in higher concentrations in bilberry dietary fibre.

### 3.2. Characteristics of Study Participants

#### 3.2.1. Anthropometric Parameters and Blood Pressure Measurements

A group of 30 overweight/obese adult women (mean age 55.83 ± 2.95 years) participated in an 8-week pre–post intervention programme with bilberry product supplementation. The women were divided into two groups: one consumed 125 mL/day of 100% organic bilberry juice, and the second consumed 10 g/day of 100% organic bilberry fibre as part of their regular diets. The parameters monitored during this intervention are shown in Table 2. Although some values of the anthropometric parameters and blood pressure changed, these changes were not statistically significant in either group.

#### 3.2.2. Changes in Basic Lipid Profile Parameters and Lipoprotein Indexes

The mean total cholesterol levels were above the reference values at the start of the study. After the 8-week intervention, a further increase was observed in both groups. In the group consuming bilberry juice, the mean TC value increased from 6.41 ± 1.23 mmol/L to 6.94 ± 1.30 mmol/L (*p* < 0.001), while in the fibre group, it increased from 6.06 ± 1.39 mmol/L to 6.43 ± 1.05 mmol/L *(p* = 0.046). Simultaneously, a statistically significant decrease in LDL-C (*p* < 0.05) and a significant increase in HDL-C (*p* < 0.001) were observed in both groups (Table 3). The TG levels in both study groups remained within the reference range, and no statistically significant changes were detected following bilberry supplementation.

In addition, there was a statistically significant improvement in the TC/HDL and LDL/HDL ratios. In the bilberry juice group, the TC/HDL ratio decreased significantly from 3.69 ± 0.92 to 3.33 ± 0.63 (*p* = 0.006), while the LDL/HDL ratio decreased from 2.15 ± 0.71 to 1.65 ± 0.49 (*p* < 0.001). Similarly, in the fibre group, there was a significant reduction in the mean TC/HDL ratio from 3.92 ± 0.90 to 3.48 ± 0.72 (*p* < 0.001), as well as in the LDL/HDL ratio from 2.34 ± 0.71 to 1.69 ± 0.49 (*p* < 0.001). However, the AIP index in both groups was not significantly affected (*p* > 0.05).

#### 3.2.3. Changes in Lipoprotein Subfractions

In the study group, the presence of atherogenic sdLDL (LDL 3-7 subfraction) was detected (using the Lipoprint^®^ system (Quantimetrix, Redondo Beach, CA, USA) before the intervention) in 12 participants in the juice group and 9 participants in the fibre group (Table 4). Statistically significant changes following the bilberry intervention were observed in the bilberry juice group (*p* < 0.05).

**Figure 2:**
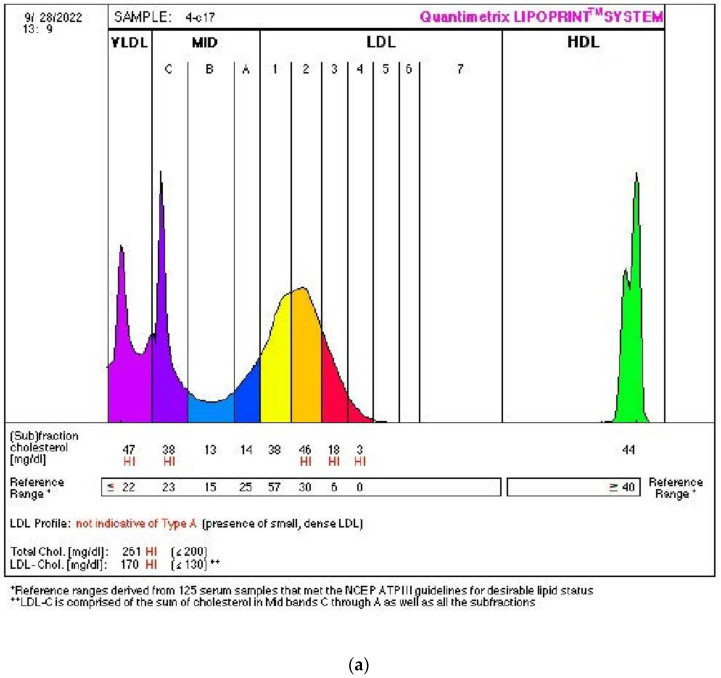

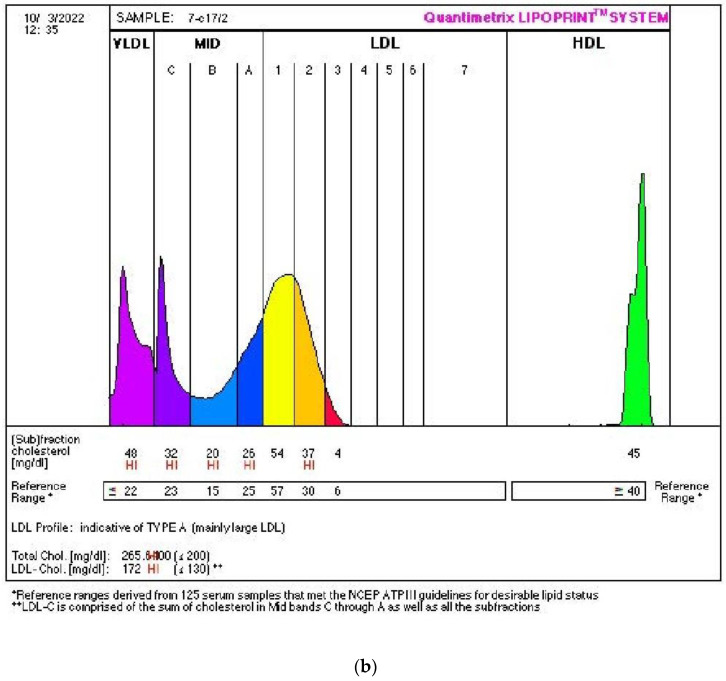
The changes in LDL subfractions in women with atherogenic phenotype B after 8 weeks of 100% bilberry juice consumption. Panel (**a**) represents the pre-intervention state characterized by LDL phenotype B, which includes atherogenic subfractions LDL3-7 (shown in red) and larger, less atherogenic subfractions LDL1-2 (shown in yellow). Panel (**b**) shows the post-intervention results, where LDL phenotype A is observed, with a significant reduction in the atherogenic LDL 3-7 subfractions (in red) and the larger, less atherogenic subfractions LDL1 (in yellow) and LDL2 (in orange). On the left, below the MID label, the IDL (intermediate-density lipoprotein) fractions A, B, C are shown in dark blue (A), light blue (B) and light purple (C). On the far left, the VLDL (very-low-density lipoprotein) value is shown in dark purple. Panel (**a**): pre-intervention, panel (**b**): post-intervention.

In response to the intervention, Figure 2 shows a typical lipoprotein profile for a woman with phenotype B. Before the intervention, the women had values for TC (6.79 mmol/L), LDL-C (4.35 mmol/L), HDL-C (1.47 mmol/L), LDL/HDL ratio (2.96 mmol/L), and LDL 3-7 (0.5439 mmol/L). These levels indicate an increased risk of CVD. The intervention with 100% bilberry juice for 8 weeks significantly improved the values of all monitored parameters: LDL-C (3.83 mmol/L), HDL-C (1.77 mmol/L), the value of the LDL/HDL ratio (2.16 mmol/L), and LDL 3-7 (0.1036 mmol/L). The TC value increased slightly (6.88 mmol/L) due to the increase in HDL-C.

## 4. Discussion

Berries have garnered increasing attention for their potential health benefits, largely due to their high content of bioactive compounds [39]. Several studies highlight the beneficial properties of bilberries (*Vaccinium myrtillus* L.) [40,41] and blueberries (*Vaccinium corymbosum* L.) [42,43], both of which are economically significant. These berries are rich in antioxidants and other beneficial compounds, which drive consumer demand and market growth despite competition from alternative crops [44].

The bilberry products used in this intervention were of commercial quality, with no added sugar, artificial sweeteners, colouring, or preservatives. The results of quantitative analyses support the presence of significant health-promoting substances in bilberries and their products, with the concentrations of total phenolics, total anthocyanins, and other selected polyphenolic compounds detailed in Table 1. The consistency of clinical evidence regarding the relationship between the consumption of fruit products, such as polyphenol-rich juices, and improved cardiovascular health has been reviewed [45,46].

The popularity of blueberries continues to grow worldwide, driven by the increasing consumption not only of the berries in fresh form but also in processed forms. The significance of blueberries for the processing industry is also evident, with the majority of blueberries being processed through freezing or juicing. Drying is also receiving attention. However, it is important to recognize that each processing method induces changes, as each step in the processing of blueberries affects the quantity and quality of biologically active compounds in different ways [47].

The analysis results reveal notable differences between the juice and fibre samples. The total phenolic content was 2.29 ± 0.01 mg GAE/mL in the juice and 32.81 ± 0.15 mg/g in the fibre. Blueberries are a rich source of anthocyanins, which constitute more than half of the total polyphenol content and are considered to have the greatest impact on consumer health [48]. In the samples used in this study, the following anthocyanin concentrations were observed: 1.48 ± 0.03 mg/L in juice and 6.82 ± 0.06 mg/g in fibre. In the study by Mendelová et al. [49], the range of anthocyanin concentrations was found to be from 1.93 g/kg to 3.95 g/kg. Wu et al. [50] found that the anthocyanin content in blueberries ranged from 2.50 g/kg to 4.95 g/kg. The lower anthocyanin concentration in the juice compared to the fibre may be attributed to the pasteurization process, which can lead to the decomposition of anthocyanins at higher temperatures during processing. Data from the study by Kalt et al. [42] also indicate that blueberry processing can change the phytochemical profile. Heat, oxygen, and enzymes can break down the phytochemicals in blueberries during processing, with the most significant losses occurring in anthocyanins and vitamin C. Berries contain vitamins A, C, and E, which act as antioxidants and can help reduce inflammation [51]. We also detected vitamin C in our samples. The fibre sample contained 1.44 ± 0.12 µg/mL vitamin C, while the juice sample contained up to 126.40 ± 0.13 µg/g. The antioxidant activity of the products was 49.30 ± 0.56% radical inhibition in the juice and 67.20 ± 0.41% in the fibre. The high antioxidant activity is attributed to the positive correlation of polyphenolic compounds present in the fruit [52,53].

Blueberries also contain other flavonoids such as catechin, caffeic acid, ferulic acid, rutin, myricetin, and quercetin, some of which were found in our samples [54,55]. According to the data, mean body weight increased slightly after the 8-week intervention compared to baseline, although the changes were not statistically significant (Table 2). Other body composition parameters also showed no significant changes. Several studies have reported similar results [20,21,56,57,58].

The effect of blueberry product supplementation on lipid profile modulation in overweight/obese women was also investigated, as shown by our results in Table 3. The lipid profile is recognized as an important risk factor and predictor of cardiovascular disease [59]. Blood serum levels of TC (juice group: *p* < 0.001; fibre group: *p* = 0.046) and HDL-C (*p* < 0.001) increased significantly after 8 weeks of supplementation. Conversely, there were statistically significant reductions in key CVD risk factors such as LDL-C (juice group: *p* = 0.010; fibre group: *p* = 0.001), while changes in TG were not statistically significant. Despite the increase in total cholesterol, short-term supplementation with bilberry products and its effects on the lipid profile in overweight/obese women are considered positive, as the increase in TC value was associated with an increase in HDL-C.

HDL-C and LDL-C play an important role in regulating total cholesterol levels in the body. Lowering LDL-C and increasing HDL-C can help reduce the risk of CVD [60,61]. LDL-C is a key parameter, and its reduction is considered the primary goal of treatment. However, despite its reduction, nearly half of the residual cardiovascular risk may persist, leading to the identification of new predictors of cardiovascular disease [62]. Several lipoprotein ratios and atherogenic indexes have been defined, and their use is justified, as they provide greater predictive power for assessing cardiovascular risk [63,64]. The TC/HDL ratio is a highly effective predictor of cardiovascular disease [35], particularly the risk of ischemic heart disease in middle-aged women and acute myocardial infarction in women aged 50–59 years [65]. The LDL/HDL ratio has also been shown to be a better indicator than either LDL or HDL alone in predicting the severity of cardiovascular disease [66]. In addition to these parameters, the atherogenic plasma index (AIP) has been proposed as a strong predictor of atherosclerosis and coronary heart disease [59]. Based on the data obtained, we calculated the TC/HDL ratio, the LDL/HDL cholesterol ratio, and the AIP index (Table 3). Although the values were consistent with reference values, they decreased after bilberry supplementation, with this decrease being statistically significant.

In addition, the presence of an atherogenic lipid profile in the subjects and the effect of blueberry supplementation on its positive modulation were identified. The atherogenic lipoprotein profile (phenotype B) is characterized by a predominance of atherogenic lipoproteins: very-low-density lipoprotein (VLDL), intermediate-density lipoproteins (IDL1 and IDL2), and, in particular, the presence of sdLDL (LDL 3-7 subfractions) [67,68].

Elevated lipoprotein levels—including very-low-density lipoprotein (VLDL), low-density lipoprotein (LDL), and small dense low-density lipoprotein (sdLDL)—have been attributed to a combination of dietary, genetic, and metabolic factors. Dietary risk factors include, in particular, a high intake of saturated fatty acids and trans fatty acids, which can subsequently lead to increased production of VLDL in the liver.VLDL is rich in triglycerides, which are then hydrolyzed by lipoprotein lipase to form intermediate-density lipoprotein (IDL) and subsequently LDL. In addition, diets high in refined carbohydrates can increase insulin resistance and further promote VLDL synthesis. There may also be a genetic predisposition. Individuals with genetic mutations or polymorphisms in genes regulating lipid metabolism such as the apolipoprotein B (apoB) and LDL receptor (LDLRs) genes may have elevated VLDL and LDL levels and a higher proportion of sdLDL due to reduced receptor-mediated clearance. Metabolic conditions, particularly insulin resistance and metabolic syndrome, are associated with increased lipolysis and free fatty acid delivery to the liver, leading to increased production of VLDL. Elevated VLDL levels contribute to increased circulating LDL particles. Insulin resistance also affects the composition of LDL particles, increasing the proportion of sdLDL, which is more atherogenic due to its tendency to penetrate the arterial wall and undergo oxidation. Consequently, the interplay of these factors engenders an environment that promotes increased levels of VLDL, LDL, and sdLDL, contributing to an increased risk of cardiovascular disease [69,70,71].

In the monitored group, the Lipoprint^®^ system detected the presence of atherogenic lipoprotein subfractions VLDL, IDL1, IDL2, and IDL3, and LDL 3-7 subfractions in 12 subjects (juice group) and 9 subjects (fibre group) before the intervention (Table 4). The positive post-intervention changes in atherogenic lipoprotein subfraction were statistically significant in the bilberry juice group (*p* < 0.05). Detecting the presence of atherogenic subfractions—i.e., atherogenic versus non-atherogenic lipoprotein profiles—represents a significant advancement in lipid diagnostics [37], which can better identify individuals at higher risk of developing CVD [72]. A significant reduction in sdLDL cholesterol particles was observed after the regular consumption of freeze-dried strawberry powder as a drink for 8 weeks [73]. A notable improvement in the atherogenic profile of overweight and obese women, with a reduction in LDL 3-7 subfractions, was observed after consuming 300 mL of berry and apple juice for 6 weeks, as reported by Habanova et al. [20]. Additionally, a reduction in small, dense atherogenic LDL cholesterol subfractions was found in men in the study by Habanova et al., which is probably related to the presence of compounds with strong antioxidant properties, such as polyphenols or vitamin C [74].

Our primary aim was not to compare two blueberry products to each other, but to determine the efficacy of these products in altering the monitored parameters of body composition and lipid profile in overweight/obese women. The interest in bilberries as a superfood is clearly based on a wealth of scientific evidence. With a wide range of therapeutic potential, this fruit could undoubtedly be innovative in the development of functional foods and nutraceuticals based on blueberries. It may also be an appropriate therapeutic strategy in managing the primary prevention of lipid profile disorders.

This study has several limitations. A key issue is the small sample size, which may have limited the statistical power to detect significant differences in outcomes, along with the relatively short duration of the intervention trial. Another limitation is the absence of a control group receiving a placebo or a pre-treatment control experiment within the same group assessing the effects of bilberry products such as 100% juice or dietary fibre. Additionally, the potential impact of thermal pasteurization on bioactive compounds, including vitamin C and certain phenolics, highlights the importance of investigating alternative pasteurization methods or untreated juice as controls. Finally, the study did not evaluate flavonoid intake from participants’ baseline diets, which could influence the observed effects.

## 5. Conclusions

Foods rich in bioactive compounds are protective in mitigating the adverse effects of risk factors associated with cardiovascular disease (CVD) development. In this study, a therapeutic intervention using bilberry-based products was introduced as part of a primary prevention strategy for CVD in overweight and obese women. While some changes were observed in body composition parameters and blood pressure, these changes were not statistically significant in either group. However, after 8 weeks of bilberry supplementation, significant improvements were noted in LDL-C and HDL-C levels, along with favourable changes in the TC/HDL and LDL/HDL ratios. In contrast, neither group detected any significant changes in TG levels. A promising outcome was the reduction in atherogenic sdLDL subfractions observed in the juice group, highlighting the potential of bilberry-based products as a non-pharmacological intervention for enhancing cardiovascular health. This is the first study demonstrating the short-term benefits of pure bilberry juice and bilberry fibre on sdLDL reduction in overweight/obese women.

These findings are clinically and commercially significant, offering potential avenues for developing novel functional products and natural approaches to CVD prevention. Future investigations should prioritize establishing the optimal dosages of products high in bioactive compounds, employing longer study durations, larger sample sizes, and the inclusion of placebo-controlled groups to enhance the reliability and applicability of the results.

## Figures and Tables

**Table 1:** The concentration of monitored biologically active compounds in bilberry juice and the dietary fibre of bilberry.

Parameters	Bilberry Juice	Dietary Fibre of Bilberry
Total phenolic content	2.29 ± 0.01(mg GAE ^1^/mL)	32.81 ± 0.15 (mg GAE ^1^/g)
Total anthocyanins	1.48 ± 0.03 (mg/mL)	6.82 ± 0.06 (mg/g)
Vitamin C	126.40 ± 0.13 (µg/mL)	1.44 ± 0.12 (µg/g)
Caffeic acid	24.87 ± 0.41 (mg/L)	85.47 ± 0.31 (mg/g)
Coumaric acid	28.59 ± 1.57 (mg/L)	125.35 ± 0.26 (mg/g)
Ferric acid	123.11 ± 0.83 (mg/L)	77.94 ± 0.21 (mg/g)
Rutin	309.95 ± 1.27 (mg/L)	24.21 ± 0.08 (mg/g)
Myricetin	34.66 ± 1.73 (mg/L)	52.79 ± 0.44 (mg/g)
Resveratrol	9.50 ± 0.74 (mg/L)	30.15 ± 0.39 (mg/g)
Quercetin	5.44 ± 0.04 (mg/L)	53.45 ± 1.05 (mg/g)
Antioxidant activity ^2^	49.30 ± 0.56 (%)	67.20 ± 0.41 (%)

Abbreviations: ^1^ total polyphenol in the samples analyzed were expressed in mg of gallic acid equivalents/mL for juice and in mg of gallic acid equivalents/g for dietary fibre. ^2^ Antioxidant activity was expressed as DPPH inhibition (%). Data are expressed as means ± standard deviation (SD).

**Table 2:** Anthropometric parameters and blood pressure measurements of volunteers of pre–post intervention of bilberry supplementation.

Parameter	100% Bilberry Juice (*n* = 15)			Dietary Fibre of Bilberry (*n* = 15)		
	Pre	Post	*p*	Pre	Post	*p*
Body weight (kg)	80.14 ± 11.86	80.17 ± 11.97	0.853	80.56 ± 8.68	81.09 ± 9.12	0.116
BMI (kg/m^2^)	29.89 ± 3.75	29.90 ± 3.81	0.842	29.52 ± 3.06	29.71 ± 3.13	0.125
WC (cm)	103.39 ± 10.49	104.21 ± 9.85	0.084	100.93 ± 7.11	101.66 ± 7.52	0.083
WHR index	1.00 ± 0.06	1.01 ± 0.05	0.073	0.97 ± 0.05	0.98 ± 0.05	0.086
SMM (kg)	24.99 ± 2.57	25.12 ± 2.51	0.151	26.76 ± 3.33	26.86 ± 3.30	0.388
FFM (kg)	45.78 ± 4.48	45.6 ± 4.41	0.334	48.59 ± 5.61	48.77 ± 5.54	0.313
FFM (%)	57.39 ± 4.10	57.66 ± 4.26	0.256	60.48 ± 4.83	60.33 ± 4.73	0.516
BFM (kg)	34.08 ± 8.40	33.98 ± 8.62	0.614	31.97 ± 5.92	32.32 ± 6.11	0.237
PBF (%)	42.44 ± 4.18	42.20 ± 4.39	0.274	39.53 ± 4.83	39.66 ± 4.74	0.561
VFA (cm^2^)	134.18 ± 26.46	134.56 ± 25.24	0.596	124.64 ± 18.99	126.59 ± 20.26	0.071
SBP (mm Hg)	126.93 ± 13.83	124.67 ± 11.73	0.271	129.80 ± 12.23	128.07 ± 11.93	0.508
DBP (mm Hg)	83.80 ± 6.93	82.20 ± 4.65	0.244	87.53 ± 6.52	86.87 ± 6.51	0.692

Abbreviations: *n*, number of participants; pre, parameters measured at baseline (before intervention); post, parameters measured after the intervention; BMI, body mass index; WC, waist circumference; WHR index, waist-to-hip ratio; SMM, skeletal muscle mass; FFM, fat-free mass (kg); FFM, fat-free mass to total mass (%); BFM, body fat mass; PBF, body fat percentage; VFA, visceral fat area; SBP, systolic blood pressure; and DBP, diastolic blood pressure. Data are expressed as means ± standard deviation (SD); statistical significance is defined as *p* < 0.05.

**Table 3:** Changes in the values of selected lipid profile parameters and lipoprotein indexes in volunteers pre- and post-bilberry supplementation intervention.

Parameter	100% Bilberry Juice (*n* = 15)			Dietary Fibre of Bilberry (*n* = 15)			
	Pre	Post	*p*	Pre	Post	*p*	RV
TC (mmol/L)	6.41 ± 1.23	6.94 ± 1.30	<0.001	6.06 ± 1.39	6.43 ± 1.05	0.046	3–5.2
TG (mmol/L)	1.34 ± 0.50	1.38 ± 0.47	0.721	1.41 ± 0.65	1.39 ± 0.68	0.880	<1.7
LDL-C (mmol/L)	3.70 ± 0.94	3.42 ± 1.00	0.010	3.56 ± 0.81	3.08 ± 0.62	0.001	<2.6
HDL-C (mmol/L)	1.78 ± 0.32	2.11 ± 0.38	<0.001	1.60 ± 0.43	1.92 ± 0.52	<0.001	>1.3
TC/HDL	3.69 ± 0.92	3.33 ± 0.63	0.006	3.92 ± 0.90	3.48 ± 0.72	<0.001	<4
LDL/HDL	2.15 ± 0.71	1.65 ± 0.49	<0.001	2.34 ± 0.71	1.69 ± 0.49	<0.001	<2.5
AIP	−0.14 ± 0.21	−0.20 ± 0.18	0.152	−0.08 ± 0.25	−0.17 ± 0.26	0.061	−0.3–0.1

Abbreviations: *n*, number of participants; pre, parameters measured at baseline (before intervention); post, parameters measured after the intervention; RV, reference value; TC, total cholesterol; TG, triglyceride; LDL-C, LDL cholesterol; HDL-C, HDL cholesterol; and AIP, atherogenic index of plasma, AIP = log(TG/HDL). Data are expressed as mean ± standard deviation (SD); statistical significance is defined as *p* < 0.05.

**Table 4:** Changes in lipoprotein subfraction values in participants with sdLDL (3-7) subfractions present before intervention.

Parameter	100% Bilberry Juice (*n* = 12)			Dietary Fibre of Bilberry (*n* = 9)		
	Pre	Post	*p*	Pre	Post	*p*
	LDL Subfractions					
VLDL (mmol/L)	1.04 ± 0.20	1.19 ± 0.25	0.011	1.12 ± 0.30	1.25 ± 0.41	0.098
IDL A (mmol/L)	0.47 ± 0.22	0.75 ± 0.18	<0.001	0.49 ± 0.27	0.60 ± 0.23	0.168
IDL B (mmol/L)	0.40 ± 0.16	0.51 ± 0.12	0.008	0.41 ± 5.15	0.51 ± 0.09	0.084
IDL C (mmol/L)	0.85 ± 0.24	0.63 ± 0.45	0.041	0.81 ± 0.23	0.70 ± 0.15	0.246
LDL 1 (mmol/L)	1.07 ± 0.32	1.35 ± 0.35	0.009	1.04 ± 0.36	1.13 ± 0.18	0.430
LDL 2 (mmol/L)	0.87 ± 0.35	0.75 ± 0.42	0.088	0.90 ± 0.28	0.84 ± 0.43	0.937
LDL 3-7 (mmol/L)	0.26 ± 0.23	0.11 ± 0.16	0.016	0.31 ± 0.32	0.24 ± 0.31	0.261

Notes: *n*, number of participants; pre, parameters measured at baseline (before intervention); post, parameters measured after the intervention; data are expressed as means ± standard deviation (SD); statistical significance is defined as *p* < 0.05.

## Data Availability

The original contributions presented in this study are included in the article. Further inquiries can be directed to the primary author on request.

## Abbreviations

The following abbreviations are used in this manuscript:

ANOVA	Analysis of variance
BFM	Body fat mass
BMI	Body mass index
CVD	Cardiovascular disease
DBP	Diastolic blood pressure
DPPH	2,2-diphenyl-1-picrylhydrazyl
EDTA	Ethylenediaminetetraacetic acid
FFM	Fat-free mass
GAE	Gallic acid equivalent
HDL	High-density lipoprotein
HDL-C	High-density lipoprotein cholesterol–refers to the amount of cholesterol carried by low-density lipoproteins in the blood
IDL	Intermediate-density lipoprotein
LDL	Low-density lipoprotein
NFC	Not from concentrate
PBF	Body fat percentage
SBP	Systolic blood pressure
sdLDL	Small dense low-density cholesterol
SSM	Skeletal muscle mass
TC	Total cholesterol
TC/HDL	Ratio of total cholesterol to high-density lipoprotein
TG	Triglyceride
VFA	Visceral fat area
VLDL	Very-low-density lipoprotein
WC	Waist circumference
WHR	Waist-to-hip ratio
